# The NTD-CTD intersubunit interface plays a critical role in assembly and stabilization of the HIV-1 capsid

**DOI:** 10.1186/1742-4690-10-29

**Published:** 2013-03-06

**Authors:** Ernest L Yufenyuy, Christopher Aiken

**Affiliations:** 1 Department of Pathology, Microbiology and Immunology, Vanderbilt University School of Medicine, A-5301 Medical Center North, Nashville, TN, 37232, USA

**Keywords:** HIV-1, Capsid, Core, Interface, Assembly, Stability, Uncoating, Reverse transcription, Crosslinking

## Abstract

**Background:**

Lentiviruses exhibit a cone-shaped capsid composed of subunits of the viral CA protein. The intrinsic stability of the capsid is critical for HIV-1 infection, since both stabilizing and destabilizing mutations compromise viral infectivity. Structural studies have identified three intersubunit interfaces in the HIV-1 capsid, two of which have been previously studied by mutational analysis. In this present study we analyzed the role of a third interface, that which is formed between the amino terminal domain (NTD) and carboxyl terminal domain (CTD) of adjacent subunits.

**Results:**

We provided evidence for the presence of the NTD-CTD interface in HIV-1 particles by engineering intersubunit NTD-CTD disulfide crosslinks, resulting in accumulation of disulfide-linked oligomers up to hexamers. We also generated and characterized a panel of HIV-1 mutants containing substitutions at this interface. Some mutants showed processing defects and altered morphology from that of wild type, indicating that the interface is important for capsid assembly. Analysis of these mutants by transmission electron microscopy corroborated the importance of this interface in assembly. Other mutants exhibited quantitative changes in capsid stability, many with unstable capsids, and one mutant with a hyperstable capsid. Analysis of the mutants for their capacity to saturate TRIMCyp-mediated restriction *in trans* confirmed that the unstable mutants undergo premature uncoating in target cells. All but one of the mutants were markedly attenuated in replication owing to impaired reverse transcription in target cells.

**Conclusions:**

Our results demonstrate that the NTD-CTD intersubunit interface is present in the mature HIV-1 capsid and is critical for proper capsid assembly and stability.

## Background

The mature HIV-1 capsid is a cone-shaped structure formed by assembly of approximately 1500 subunits of the viral CA protein into a lattice of hexamers, with pentamers closing the ends [1,2]. The capsid lattice is stabilized by intersubunit interactions within and between hexamers. The amino-terminal and carboxyl-terminal domains (designated NTD and CTD, respectively) of CA are predominantly alpha-helical and fold independently [3,4]. Recent structural studies have provided a detailed model of the capsid and have defined protein interfaces in the NTD and CTD through which the subunits interact [5-8]. These interfaces include a six-fold intra-hexameric NTD-NTD interface [6,8]; a dimeric inter-hexamer interface formed between CTDs [3,5,6,9]; and a CTD-CTD trimeric interface that connects the hexamers [5]. The structures have also revealed the presence of an NTD-CTD intersubunit interface between adjacent CA subunits within the hexamers [8].

The viral capsid plays a critical role in early events in the HIV-1 life cycle. Following fusion of the HIV-1 particle with a susceptible host cell, the capsid undergoes disassembly from the ribonucleoprotein complex in a process termed uncoating. Uncoating is a poorly understood stage in the HIV-1 life cycle. Mutagenesis studies have indicated that subunit interactions in the capsid lattice confer specific intrinsic stability to the HIV-1 capsid. Mutations at the NTD-NTD and CTD-CTD intersubunit interfaces affect capsid stability [5,10-12]. CA mutants with reduced capsid stability often exhibit impaired infectivity, suggesting that the intact capsid performs a critical function following penetration into the target cell. Imaging studies of HIV-1 CA mutants in target cells further provided evidence for a role of reverse transcription in facilitating uncoating, suggesting that deviations in capsid stability might affect reverse transcription [13,14]. Furthermore, capsid-targeting cellular restriction factors that block infection at an early phase of replication appear to act by inducing premature uncoating in target cells [15-17]. Mutagenesis studies have also shown that CA surfaces are important for both particle assembly and capsid formation, and CA mutants that exhibit abnormal core morphology are poorly infectious [18-22]. Thus, the emerging view is that the capsid is a critical component of the virion whose structure, assembly, and disassembly require a better understanding for antiviral targeting. Besides CA, other Gag components, including NC, are also important for shaping the structure of the virion [23].

NTD-CTD intersubunit interactions were initially suggested by *in vitro* kinetic assembly studies of full-length CA protein and truncated N-terminus and C-terminus proteins [24]. This initial suggestion was further enhanced by observations that hydrogen-deuterium (H/D) exchange on *in vitro* assembled CA occurred slowly at the NTD and at the CTD regions suggesting that these regions are protected. Chemical crosslinking and high-resolution mass spectrometry in the same study showed that Lys 70 on one subunit is in proximity to Lys 182 on a different CA subunit [25]. This interface was later visualized in atomic resolution in the crystal structure of a CA hexamer [8]. Structural and genetic studies of other retroviruses, specifically RSV and SIV (both showing second-site suppressors), also indicated the presence of an interdomain interaction in the capsid involving the NTD of one subunit and the CTD of an adjacent subunit [26-28]. This is consistent with the overall structural conservation exhibited by retroviral CA proteins [29-33].

In HIV-1, the NTD-CTD interface is formed by helices 4 and 7 on the NTD of one subunit and helices 8 and 11 on the CTD of the adjacent subunit [6,8]. The NTD-CTD interface is present in assemblies of recombinant CA and in disulfide-stablized CA hexamers and pentamers; furthermore, studies of HIV-1 virus-like particles by H/D exchange demonstrated that peptides mapping to this region of the CA-NTD were protected from solvent, suggesting that the interface is present within the viral capsid [34]. Nonetheless, neither the structure of the interface in the context of mature HIV-1 particles, nor its role in HIV-1 replication, has been studied in detail.

To investigate the role of the NTD-CTD intersubunit interface in capsid assembly and stability, we used the crystal structure of the HIV-1 CA hexamer as a guide to design a panel of HIV-1 mutants encoding substitutions in the NTD-CTD interface. We confirm via engineered disulfide crosslinking that the NTD-CTD interface is present within HIV-1 particles. Analysis of a panel of single amino acid substitution mutants for capsid functions *in vitro* and *ex vivo* revealed that the interface is critical for HIV-1 capsid structure and stability, and for viral infectivity**.**

## Results

### Engineered cysteine substitutions at the NTD-CTD interface result in spontaneous disulfide crosslinking of CA in virions

**Figure 1:**
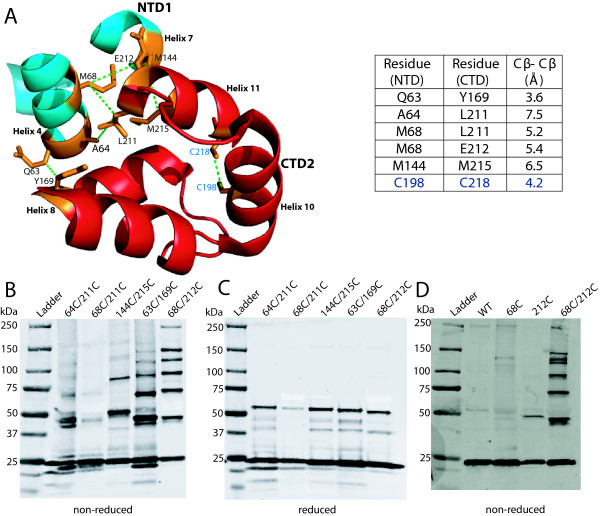
**CA subunits virions with engineered cysteines at the NTD-CTD capsid interface spontaneously crosslink into hexamers in HIV-1 particles.** (**A**) A view of the NTD-CTD interface showing the NTD helices 4 and 7 (blue) from one subunit in close proximity to the CTD helices 8 and 11 (red) from another subunit (PDB: 3H4E). Residues mutated to cysteines for crosslinking are shown in orange and are connected by green dotted lines. The two endogenous Cys are shown for comparison. Distances in angstroms (Å) from a Cβ-carbon of one amino acid residue on NTD1 of one subunit to a Cβ-carbon on a second amino acid residue on CTD2 from a different subunit are shown. The Cβ-Cβ distance between endogenous cysteines is shown for comparison. (**B**, **C**, and **D**) Immunoblot analysis of viral lysates. Pelleted particles were dissolved in 1X SDS sample buffer in the absence (**B**) or presence (**C**) of β-mercaptoethanol. Samples were then separated by SDS-PAGE and analyzed by immunoblotting with CA-specific antibody. (**D**) Analysis of the single mutants M68C and E212C under non-reducing conditions.

The X-ray structure of the HIV-1 CA hexamer capsid revealed the presence of an intermolecular interface between the NTD of one CA subunit and the CTD of the adjacent subunit [8]. To test whether the NTD-CTD interface is detectable within the viral capsid lattice, we generated full-length HIV-1 molecular clones encoding double Cys substitutions across the interface. Using the structural display program Pymol, we quantified distances between interface residues in the X-ray structure of the HIV-1 CA hexamer (PDB code 3H4E, Figure 1A). Distances within ~5 Å (Cβ-Cβ) were targeted. We reasoned that if the interface is present within the viral mature capsid, adjacent subunits with mutant cysteine residues should form spontaneous disulfide crosslinks. In a previous study, we had successfully employed this approach to provide evidence for the existence of a CTD-CTD trimeric interface in mature HIV-1 particles [5]. We engineered a total of 5 Cys pairs spanning the interface (Figure 1). The mutant viruses were produced by transfection of 293T cells with the mutant plasmids, pelleted, and analyzed for spontaneous CA-CA crosslinking by non-reducing SDS-PAGE and immunoblotting. When tested on TZM-bl cells, the double Cys mutants were not infectious (data not shown). The A64C/L211C and M68C/L211C mutants did not form crosslinked CA bands higher than dimers (Figure 1B), but the M144C/M215C mutant exhibited dimer and trimer CA species, possibly as a result of geometric constraints. Q63C/Y169C and M68C/E212C particles exhibited ladders of oligomers corresponding to dimers, trimers, tetramers, pentamers, and hexamers (Figure 1A). Of the mutants, M68C/E212C exhibited the greatest extent of crosslinking. Upon treatment with reducing agent, all higher CA bands of the double mutants were converted into the monomeric form (Figure 1C), demonstrating that the oligomeric species were stabilized by disulfide bond formation. When M68C/E212C virions were analyzed by transmission electron microscopy (TEM), the particles exhibited a variety of shapes and sizes, suggesting that these residues may be involved in the initial assembly of the immature particle. The capsids varied in size and shape, but still exhibited spherical and conical structures (data not shown). Regarding the M68C/E212C mutant, analysis of virus mutants containing either individual cysteine substitution revealed that crosslinking was dependent on the presence of both substitutions (Figure 1D). Collectively, these data confirm that the NTD-CTD intersubunit interface is present within the hexameric unit in the capsid lattice. The formation of hexameric crosslinking within the mutant virions suggested that the NTD-CTD interface is likely to play an important role in capsid structure and function.

### NTD-CTD interface substitutions impair HIV-1 replication in T cells

**Figure 2:**
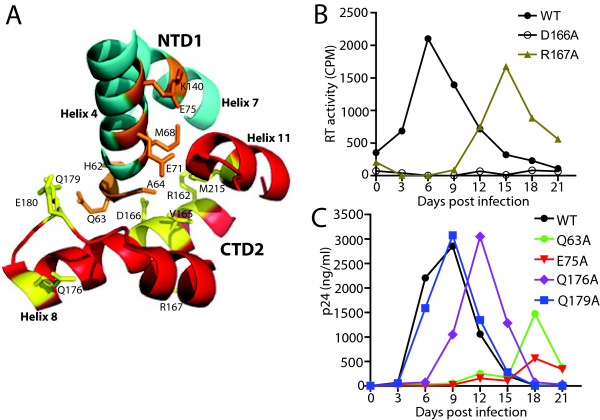
**Impaired replication of NTD-CTD mutants in CEM cells.** (**A**) Structural view of the NTD-CTD interface showing two different subunits, NTD1 (blue) and CTD2 (red) and the side chains of residues target for mutagenesis. (**B** and **C**) CEM cells were inoculated with 5 ng of virus or an equivalent quantity based on RT units. Supernatants were sampled from the culture media after every 3 days as indicated and viral accumulation analyzed by p24 ELISA or by *in vitro* RT assay. The results shown are the mean values of duplicate determinations.

To study the role of the NTD-CTD interface in HIV-1 capsid structure and function, we targeted amino acids at positions in the interface for mutagenesis. To this end, HIV-1 proviruses containing single substitutions in the NTD and CTD were produced. The mutants included fourteen alanine substitutions and one aspartic acid substitution (Table 1). Figure 2A shows a view of the interface in the crystal structure, showing each position that was targeted. To characterize and evaluate the effects of the mutations on HIV-1 replication, we quantified virus accumulation in cultures of the human CEM T cell line. Cultures were inoculated with wild type and mutant viruses at low MOI, and the cultures were monitored for the accumulation of p24 or RT activity (for mutants not recognized by our ELISA primary antibody) in the culture supernatants. As expected, the wild type virus replicated efficiently in CEM cultures, reaching peak levels at day 6–9 days post-inoculation. By contrast, the Q63A, E75A and R167A mutants were markedly delayed in replication, peaking at days 18 or 15, and exhibiting maximum p24 levels lower than that of the wild type (Figure 2B and 2C). The delayed replication of the mutants may have been due to reversion or accumulation of additional mutations, though this was not specifically examined. The duplicate cultures exhibited similar results, arguing against pseudoreversion, which would likely be stochastic. Q176A was delayed by 3 days, but reached a similar peak level as the wild type (Figure 2C). The Q179A mutant replicated with nearly wild-type kinetics (Figure 2C). However, the remaining ten mutants failed to replicate, including five NTD substitutions (H62A, A64D, M68A, E71A, K140) and five CTD mutants (R162A, V165A, D166A, E180A, M215A) (data not shown). Hence, most changes at the NTD-CTD interface resulted in severe impairments in replication in primary T cells.

**Table 1:** Summary of viral mutant phenotypes

**Virus**	**Mutation Location**	**Single-cycle infectivity (% of WT ± SD)**	**Replication in CEM cells**^**@**^	**% of mature cores**^**#**^	**Presence of cones/tubes**	**% core-associated CA (±SD)**	**Disassembly *in vitro***	**Abro-gation of restriction**	**Reverse transcription in cells (% of WT ± SD)**
WT	-	100	+++	50	+	14 ± 2	Normal	Yes	100
H62A	Helix 4	1.0 ± 0.3	-	10	-	1.1 ± 0.4	ND	No	1.3 ± 0.5
Q63A	Helix 4	27 ± 3	+	15	+	5.2 ± 0.2	Accelerated	Yes	50 ± 14
A64D	Helix 4	0 ± 0.1	-	35	-	1 ± 0.6	ND	No	1.3 ± 0.5
M68A	Helix 4	1.0 ± 1	-	10	-	0.8 ± 0.3	ND	No	1.3 ± 0.5
E71A	Helix 4	18 ± 4	-	20	+	6.3 ± 2	Normal	Yes	49 ± 6.2
E75A	Helix 4	13 ± 3	+	20	+	3.6 ± 1	Accelerated	Yes	34 ± 6.2
K140A	Helix 7	1.0 ± 0	-	20	-	0 ± 0	ND	No	1.0 ± 0.8
R162A	Helix 8	0 ± 0.2	-	35	+	0.2 ± 0.1	ND	No	1.7 ± 0.5
V165A	Helix 8	1.0 ± 1	-	25	*a*	31 ± 4	Slowed	Yes	2.0 ± 0.8
D166A	Helix 8	1.0 ± 1	-	35	-	1 ± 0.1	ND	No	1.3 ± 0.5
R167A	Helix 8	11 ± 2	+	35	+	6.4 ± 1.1	Accelerated	No	32 ± 5.2
Q176A	Helix 8	25 ± 3	++	30	+	8.1 ± 2	Normal	Yes	83 ± 13
Q179A	Helix 8	62 ± 12	+++	35	+	13 ± 2	Normal	Yes	94 ± 7.9
E180A	Helix 8	4 ± 1	-	20	-	5.1 ± 1.4	Normal	Yes	30 ± 1.7
M215A	Helix 11	0 ± 0.3	-	20	-	0.5 ± 0.1	ND	No	1.5 ± 0.5

^@^ +++, replicates like wild type; ++, 3 days delayed; +, 15–18 days delayed; -, no replication within 30 days.

^#^ mature cores exhibit an electron-dense core region, which is either bar-shaped, conical or spherical.

ND; not determined.

*a*: conical but morphologically aberrant cores.

### Most changes in the NTD-CTD interface do not affect HIV-1 particle production

**Figure 3:**
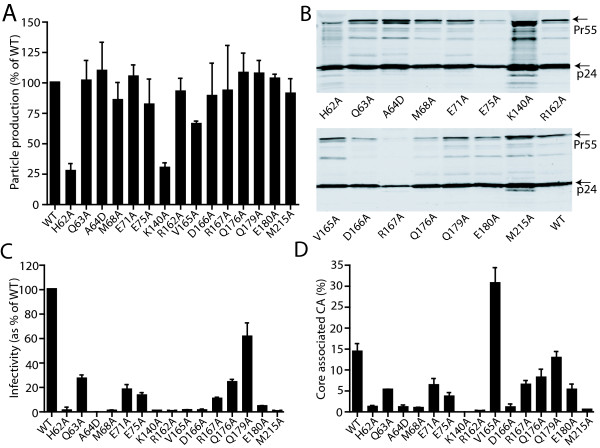
**Mutations at the NTD-CTD intersubunit interface impair HIV-1 infectivity and alter capsid stability.** (**A**) Production of viral particles as measured by RT activity. (**B**) Immunoblot analysis of viral lysates. Virions were collected from transfected 293T cells, pelleted, and analyzed by SDS-PAGE and immunoblotting with a CA specific polyclonal antibody. (**C**) Single-cycle infectivity of the mutant virions. Virion stocks were titrated on TZM-bl reporter cells and the extent of infection was determined by quantification of luciferase reporter activity in cell lysates. Values were normalized by the corresponding values of reverse transcriptase activity in the inocula. Results shown are the mean values of three independent experiments, with error bars representing one SD. (**D**) Quantification of core-associated CA. Cores were purified from the concentrated virions, and the levels of the core-associated CA were determined as a percentage of the total virion-associated CA. The values shown are the means of three independent experiments; error bars represent one SD.

Substitutions in CA can inhibit particle production by perturbing lattice interfaces, altering Gag cleavage, or inhibiting protein folding or stability [35-38]. To assess the effects of NTD-CTD interface substitutions on particle production, we transfected 293T cells and determined the quantity of viral proteins released into culture supernatants by RT activity assays. In most cases, particle production was similar to that of the wild type, except for H62A and K140A, which exhibited reduced particle production (Figure 3A). The reduced particle yield of the latter two mutants was not due to poor expression of Gag in the producer cell, as determined by immunoblotting analysis of lysates of the transfected cells (data not shown). To determine whether Gag processing was altered by the mutations, we pelleted the viral particles and analyzed the viral proteins by SDS-PAGE and immunoblotting with a CA-specific antibody. Most of the mutants exhibited a normal banding pattern, suggesting that the observed replication defects were not caused by aberrant processing of Gag precursors. Exceptions were the H62A, K140A and M215A mutants, which exhibited additional CA-reactive bands at ≤23kD (Figure 3B). The precise source of these bands was not determined. Conceivably, these three mutations might have altered the tertiary and quaternary structure of the CA protein, such that sites not normally cleaved by proteases became exposed to enzyme activity resulting in non-canonical cleavage. Because proteolytic processing of Gag is initiated during HIV-1 particle assembly, the aberrant cleavage could also contribute to the impaired particle production observed for the H62A and K140A mutants (Figure 3A).

To determine the consequences of NTD-CTD interface mutations on HIV-1 infectivity, we titrated the particles on TZM-bl indicator cells. Cells were lysed and quantified for luciferase activity, which was normalized by the input levels of RT activity in the inocula. A majority of mutants including Q63A, E71A, E75A, R167A, Q176A, E180A exhibited moderate-to-severe reductions (~10-30% of WT) in infectivity (Figure 3C). Infectivity was virtually abolished (<2% of WT) by the mutations H62A, A64D, M68A, K140A, V165A, D166A, M215A, while the infectivity of the Q179A mutant was similar to that of the wild type. These results suggest that the impaired replication by the mutant viruses is due to an early defect in the viral life cycle, since viral production after transfection was unaffected, except for the two aforementioned mutants (H62A and K140A).

### Point mutations at the NTD-CTD interface alter the level of CA associated with purified HIV-1 cores

The HIV-1 capsid is a metastable structure, and mutations in CA that alter the intrinsic stability of the capsid are generally deleterious to infectivity [5,10,11]. The X-ray crystal structure of the CA hexamer suggested that NTD-CTD intersubunit interactions likely contribute to capsid lattice stability [8]. To test this hypothesis, we purified viral cores from the mutant particles and quantified the percentage of CA cosedimenting with the cores. For the wild type, a mean value of 14.3% was obtained from three independent experiments. This value is consistent with previous reports that only a minor fraction of CA in virions participates in capsid assembly [5,11,14,39-41]. By contrast, a majority of the NTD-CTD interface mutants exhibited levels of core-associated CA lower than that of the wild type (Figure 3D). Based on the results, we categorized the mutants as follows: hyperstable (V165A); moderately reduced stability (Q63A, E71A, E75A, R167A, Q176A, E180) and highly unstable (H62A, A64D, M68A, K140A, R162A, D166A, M215A) (Table 1). Importantly, the alterations in core-associated CA were correlated with the impaired infectivity for the mutant viruses (Figure 3C).

### Cores from a subset of NTD-CTD interface mutants exhibit altered rates of uncoating *in vitro*

**Figure 4:**
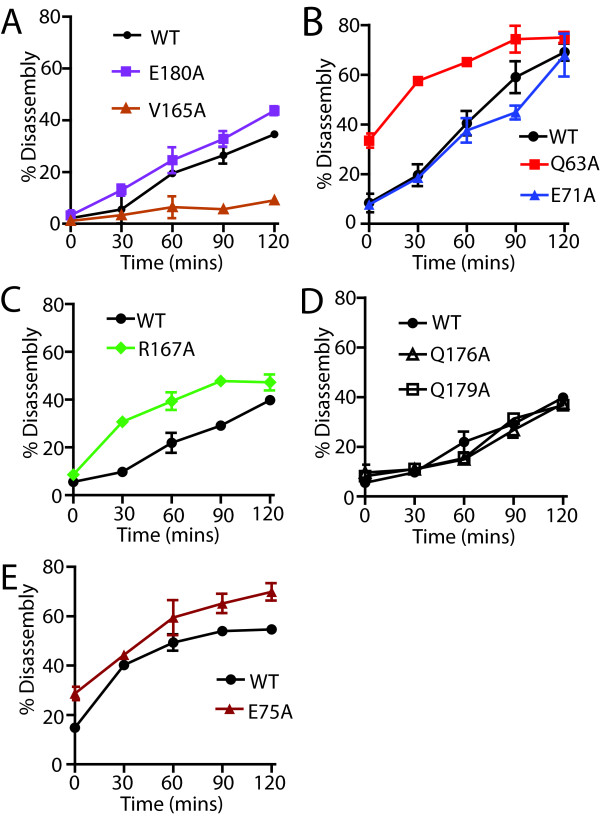
**NTD-CTD CA mutant cores undergo accelerated uncoating*in vitro*.** Purified HIV-1 cores were diluted in STE buffer and incubated at 37°C for the indicated times. After pelleting the particles, the extent of uncoating was determined by quantifying CA levels in the pellets and supernatants. Note that variations in uncoating can occur from batch-to-batch isolated cores; hence for each panel analyzed, cores were isolated at the same time. Shown are the mean values of duplicate determinations, with error bars representing the range of values. The results are representative of a minimum of two independent experiments for each mutant.

To further analyze the effects of the mutations on HIV-1 capsid stability, we quantified the uncoating of cores purified from the Q63A, E71A, E75A, V165A, R167A, Q176A, Q179A, and E180A mutants. Samples of purified cores were diluted into buffer and incubated at 37°C for various time periods, after which the cores were pelleted and the extent of uncoating determined by quantifying the CA present in the supernatant and pellet. We observed altered rates of uncoating for a subset of the mutants. Cores from the Q63A, E75A, and R167A mutants uncoated more rapidly than the wild type, while E71A, Q176A and Q179A cores uncoated with kinetics similar to the wild type (Figure 4A-C). E180A cores reproducibly uncoated slightly faster than wild type cores in three independent experiments. By contrast, V165A cores uncoated more slowly than the wild type (Figure 4A), in concordance with the elevated level of core-associated CA exhibited by this mutant (Figure 3D). Based on the collective results shown in Figure 3D and Figure 4, we conclude that a majority of mutations in the NTD-CTD interface destabilize the viral capsid, while the V165A renders the capsid hyperstable (summarized in Table 1).

### NTD-CTD interface mutants exhibiting highly unstable capsids are impaired for abrogation of restriction by TRIMCyp

**Figure 5:**
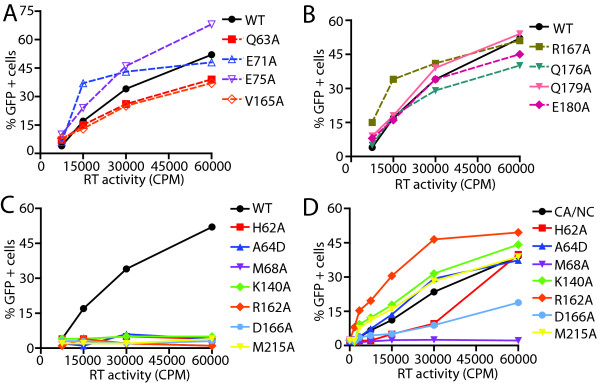
**NTD-CTD CA mutant capsids fail to abrogate rest riction by TRIMCyp.** OMK cells were co-inoculated with fixed amount (5 ng) of HIV-GFP and the indicated quantities of CA mutant viruses. (**A**) and (**B**) are separated for clarity, while (**C**) and (**D**) show abrogation activity for the mutants in the wild type and the CA-NC cleavage mutant background, respectively. The values shown are the average of duplicate determinations and are representative of two independent experiments.

TRIM5 host restriction factors inhibit retrovirus infection by inducing premature uncoating in target cells [17,42-44]. Restriction by these factors is saturable and can be overcome *in trans* by virus-like particles. We have shown that saturation of restriction is dependent on the stability of the HIV-1 capsid [39,45]. Thus, the ability of HIV-1 particles to enhance infection of an HIV-GFP reporter virus *in trans* can be a useful probe of HIV-1 uncoating in target cells. To probe the stability of the NTD-CTD interface mutants *in vivo*, we quantified their ability to enhance infection by an HIV-1 reporter virus in OMK cells, which endogenously express TRIMCyp. CA mutants with moderately reduced capsid stability (Q63A, E71A, E75A, R167A, Q176A, Q179A, E180A), as well as the hyperstable mutant V165A, enhanced infection of HIV-GFP virus *in trans* (Figure 5A and 5B). By contrast, mutants with highly unstable capsids (H62A, A64D, M68A, K140A, R162A, D166A and M215A) were markedly impaired in their ability to overcome restriction by TRIMCyp (Figure 5C). These results suggested that the NTD-CTD mutants with unstable capsids undergo premature uncoating in target cells.

Mutations in CA can compromise the ability of the virus to engage TRIMCyp either directly by altering recognition, or indirectly by inducing premature uncoating. We previously demonstrated that the addition of mutations preventing cleavage between CA and NC can rescue the ability of mutants with unstable capsids to overcome restriction, most likely by stabilizing the capsid [39,45]. Although it seems unlikely that NTD-CTD interface substitutions would directly alter recognition by TRIMCyp, which binds to an exposed CA loop on the outer surface of the capsid, we nonetheless analyzed mutants containing both the NTD-CTD substitutions together with the CA-NC cleavage site mutants. In this context, all of the unstable mutants except M68A enhanced infection by HIV-1 GFP (Figure 5D). Thus for the majority of the unstable mutants, the failure to abrogate restriction in OMK cells can be attributed to the instability of their capsids and not to a direct effect on the CA substitutions on recognition by TRIMCyp. These data support the conclusion that NTD-CTD interface residues contribute to proper uncoating in target cells.

### Mutations in the NTD-CTD interface impair HIV-1 reverse transcription in target cells

**Figure 6:**
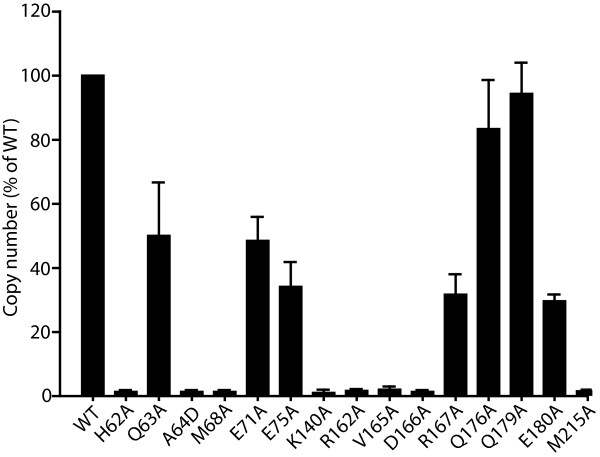
**NTD-CTD CA mutants with altered capsid stability are impaired in reverse transcription in target cells.** HeLa-P4 cells were inoculated with equal quantities of WT or mutant viruses. Cultures were harvested 8 hr. after infection and DNA isolated and assayed for second-strand transfer products by qPCR. Shown are the mean values and error bars represent the standard deviations from three independent experiments.

The majority of the NTD-CTD interface mutants released normal quantities of particles that were poorly infectious, indicative of an early post-entry defect. Previous studies from our laboratory and others have shown that capsid-destabilizing mutations are often associated with impairments in reverse transcription [10,18,46]. Therefore, we employed stage-specific PCR to quantify the ability of the NTD-CTD capsid mutants to undergo reverse transcription in target cells. The mutants exhibiting altered capsid stability displayed markedly reduced accumulation of late reverse transcripts *in vivo* (Figure 6). Additional experiments revealed that the mutants also produced reduced levels of early products (full-length minus strand; data not shown). Our results show that the reduced infectivity of the NTD-CTD interface mutants is associated with impaired reverse transcription. Reverse transcription appeared to be correlated with the degree of capsid stability, as partially unstable CA mutants accumulated less DNA compared to WT, and no DNA products were detected for highly unstable mutants (Figure 6). The hyperstable mutant V165A was also impaired for reverse transcription in target cells.

To determine whether the decrease in reverse transcription of the CA mutants in target cells is due to a decrease in virion-associated RT activity, we assayed virus stocks for exogenous RT activity and for CA antigen in parallel. The results of the two assays were concordant, indicating that the CA substitutions did not affect the levels of active RT enzyme within the particles (Figure 3 and data not shown).

### Ultrastructural analysis of NTD-CTD mutant virions

**Figure 7:**
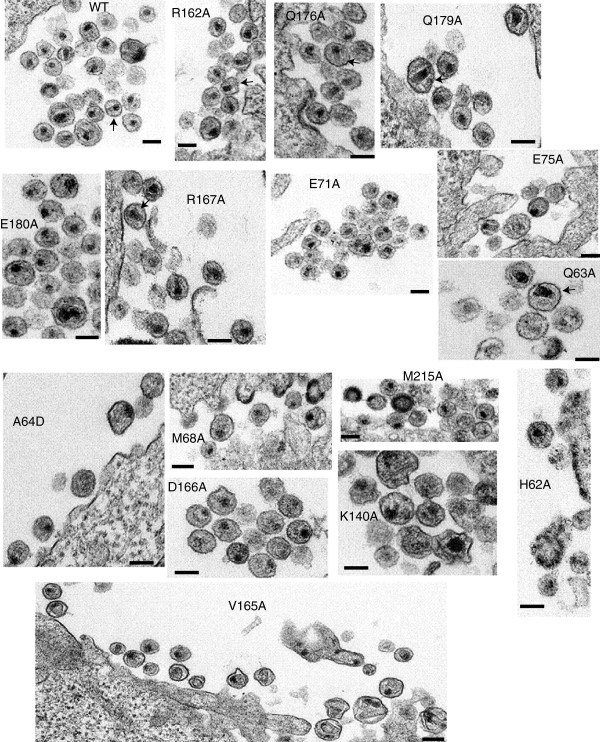
**Electron microscopy of NTD-CTD interface mutants.** HeLa cells were transfected with R9 plasmids containing either WT or alanine mutations. 24 hr post transfection, the cells were fixed in buffer containing 2.5% glutaraldehyde. Following embedding and staining, ultrathin sections were then examined in a FEI Tecnai Spirit Twin transmission electron microscope. Scale bars represent 100 nm Arrows indicate particles with conical capsids.

**Figure 8:**
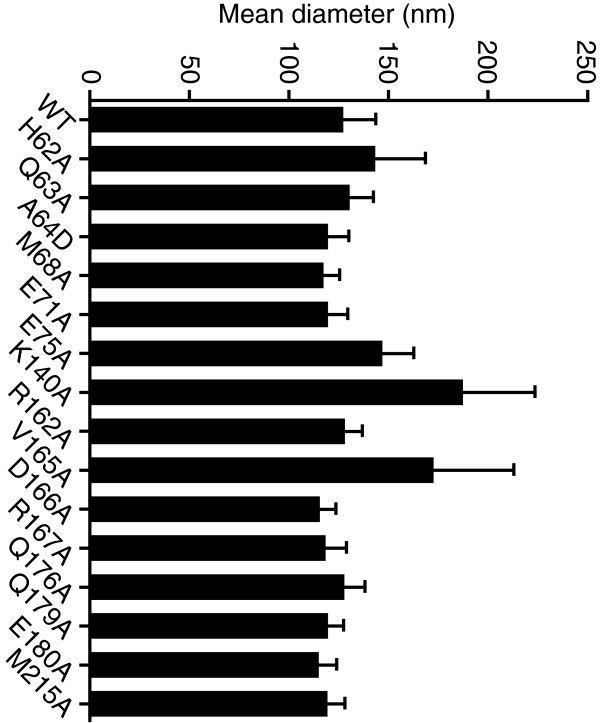
**Effects of NTD-CTD interface substitutions on HIV-1 particle size.** The diameter of HIV-1 particles was quantified. Shown are the mean values, with error bars represent one standard deviation.

To examine potential effects of the NTD-CTD interface substitutions on core structure, we performed ultra-thin section transmission electron microscopic (TEM) analysis of WT and mutant virions produced from transfected HeLa cells. Particles associated with cells transfected with the wild type HIV-1 proviral construct exhibited various stages of morphogenesis, including budding particles and immature and mature particles, with mature particles exhibiting conical, tubular, and spherical capsids near the cell surface, depending on the plane of sectioning (Figure 7). The conical and spherical capsids also contained an electron-dense nucleoid typical of the HIV-1 ribonucleoprotein complex. Immature particles were apparent in both the wild type and CA mutants, but occurred at a slightly higher frequency for the mutants (Table 1). Analysis of size of the particles demonstrated that most of the mutations did not alter the diameter of the particles, with three exceptions (Figure 8). Statistical analysis of the results indicated that E75A, K140A, and V165A each exhibited slightly elevated virion diameters compared to wild type. The E75A mutation was previously associated with altered particle size, as the double mutant E75A/E76A exhibited enlarged particles [20]. A study of HIV-1-RSV chimeric viruses also observed a link between CA and the size of immature particles [33].

With respect to capsid morphology, cones and tubes were also apparent in six of the mutants (Q63A, E75A, R162A, R167A, Q176A, Q179A) (Figure 7). However, seven of the mutants (H62A, A64D, M68A, K140A, D166A, E180A and M215A) lacked the conical morphology of the wild type (Figure 7) but retained the electron-dense internal structure of the RNP complex. E71A particles exhibited multiple core-like internal structures without clear conical capsids. V165A mutant particles appeared pleomorphic, with the internal capsid exhibiting spheres and cones varying greatly in size and shape. Overall, the effects of the mutations on capsid morphology suggest that residues at the NTD-CTD interface are important for proper capsid structure as well as stability.

## Discussion

Our previous studies identified side chains in the NTD and CTD that are critical for HIV-1 capsid stability [11,47]. At the time of those studies, the positions of the mutations in the CA hexamer were not known, as the high-resolution structure of the hexamer had not yet been reported. Subsequent studies showed that several of the NTD substitutions that perturbed capsid stability (P38A, E45A, T54A) were located at the NTD-NTD interface [8], while some of the CTD substitutions (K203A, Q219A) were present at the 3-fold CTD-CTD interhexameric interface [5]. In the present study, we focused on the role of the NTD-CTD intersubunit interface, which was not well characterized at the time of the previous studies, in HIV-1 capsid structure and function.

High-resolution structural studies provided the foundation for this investigation. The CA hexamer structure suggested that the NTD-CTD interface is likely to be important for either capsid assembly or stability. Previous structural and biochemical studies of HIV-1 and other retroviral capsids demonstrated that this interface is present in the capsid lattice formed by assembly of CA *in vitro*[6,8,24-28,34]. However, the role of the NTD-CTD interface in the virion and its contribution to capsid function had not been demonstrated.

Our engineered disulfide crosslinking data confirms that the NTD-CTD interface is a component of the native HIV-1 capsid lattice. Crosslinking between Cys residues at positions 68 and 212 resulted in the formation of monomer, dimer, trimer, tetramer, pentamer and hexamer bands. Lack of crosslinked CA species in the single mutants (M68C and E212C) showed that the banding results from disulfide bonding between the engineered cysteines (Figure 1D). Further evidence for the NTD-CTD hexameric interface was also provided by crosslinking observed in the Q63C/Y169C, A64C/L211C, M144C/M215C and M68C/L211C mutants, though at reduced efficiency (Figure 1B), which was not enhanced upon chemical oxidation with copper *O*-phenanthroline (our unpublished observations). Engineered crosslinking provides an experimental probe for the formation of an NTD-CTD interface in mutant virions. We have recently employed this approach to study the formation of the individual capsid intersubunit interfaces during maturation [48], and it may also prove useful for studying the effects of small molecules on capsid assembly and structure.

We also observed that amino acid substitutions at the NTD-CTD interface affect capsid assembly. Three of the mutants (A64D, M68A, D166A) exhibited normal Gag processing, and produced particles at wild type levels. However, the virions had significant reductions in the number of conical capsids (Figure 7, Table 1). D166 is an interdomain helix-capping amino acid, whose interactions with helix 4 of an adjacent subunit provides an amino terminal capping for the NTD-CTD interaction [8]. Other substitutions at the interface, including H62A, K140A and M215A, also perturbed capsid structure. However, these three substitutions led to altered processing of Gag, suggesting that the changes may affect capsid structure through maturation defects. A previous study of H62A had also reported that this mutant is impaired for HIV-1 capsid assembly [19]. H62 occupies the hydrophobic core of the NTD-CTD interface forming stacking interactions with F32 and Y145 [6,8]. The phenotype of the H62A mutant is also reminiscent of the effects of CAP-1, a small molecule capsid assembly inhibitor that binds the NTD pocket and disrupts the packing of the Phe32 and Tyr145 aromatic side chains [8,49]. K140 and M215 lie in close proximity to the helix-capping amino acids R143 and Q219, respectively [8]. Their proximity to helix-capping amino acids suggest that substitutions at these positions may alter contacts at the NTD-CTD interface and perturb capsid assembly. Previous work from another group had shown that the NTD-CTD interface substitutions Y169A and L211A also induced severe Gag processing defects [35], which may result from the generation of novel cleavage sites or from structural alterations induced by the mutations.

A major finding from this work is that substitutions at the NTD-CTD interface alter capsid stability (Table 1). Most of the substitutions at the NTD-CTD interface produced virions with unstable capsids, all of which were poorly infectious. The mutations that destabilized the capsid *in vitro* also resulted in accelerated uncoating in target cells, as indicated by the reduced ability of the mutant particles to abrogate restriction by TRIMCyp. The *in vitro* uncoating data agreed well with the abrogation of restriction results. However, Q63A, R167A, and E180A were exhibited rapid uncoating *in vitro*, but these mutants abrogated restriction with wild type efficiency. Our understanding of the mechanism of restriction abrogation is limited; however, we have shown that addition of a second-site suppressor mutation restores the abrogation activity of the P38A mutant without reversing its intrinsic capsid instability [14], suggesting that mutations can perturb uncoating in target cells without altering the biochemical stability of the capsid. The Q179A substitution also had no apparent effect on capsid stability or viral infectivity. Structurally, Q179 is located at the end of helix 8 and is near N57, Q63 and M66 in the NTD. Currently, it is not clear why the Q179A substitution was not deleterious to capsid function; we surmise that the location of this side chain near the edge of the interface may be a factor.

The R162A mutant exhibited conical capsids that were unstable (Figure 3D and Figure 5C). The mutant was impaired for reverse transcription in target cells (Figure 6), was poorly infectious and was impaired for abrogation. We attribute the phenotype of this mutant to a capsid stability defect. Structurally, R162 is near the stack formed by Y145, H62 and F32; the R162 guanidinium group is thought to stabilize the stacking of these aromatic side chains [8]. It is plausible that the substitution of alanine at this position changes the juxtaposition of the Y145 and F32 side chains at the NTD-CTD interface, thus altering the structure at the interface and rendering the capsid unstable.

In an earlier study we reported that E45A exhibited an impairment in reverse transcription in target cells [10]. More recently, we observed that this mutant is, in fact, competent for reverse transcription in target cells [14]. In the present work, we observed that the hyperstable V165A mutant is impaired for reverse transcription, like the unstable mutants (Figure 6). Preliminary analysis of the cores purified from V165A by immunoblotting revealed that the mutant cores are enriched in unprocessed Gag and Gag-Pol (our unpublished observations). We speculate that the presence of these intermediates prevented reverse transcription by interfering with the activity of the processed RT or by perturbing capsid structure.

Our results highlight the NTD-CTD intersubunit interface as a potentially attractive target for therapeutic intervention. Recently, small molecule inhibitors targeting HIV-1 CA have been identified [50-53]. One of these, PF-3450074 (PF74), binds to a pocket in the NTD located near the NTD-CTD interface and destabilizes the capsid [41,52]. Substitutions in the interface can lead to resistance by blocking the binding of PF74 [41], but these mutations may also result in reductions in viral fitness. Another small molecule, CAP-1, has been shown to alter subunit interactions at the NTD-CTD interface [8,49,53]. Our finding that many NTD-CTD interface mutants exhibit impaired replication suggests that inhibitors targeting this interface may exhibit a high barrier to emergence of resistance. Further efforts to design capsid-targeting HIV-1 inhibitors will benefit from a thorough understanding of the structure and function of the viral capsid.

## Conclusions

Our results show that the NTD-CTD intersubunit interface is critical for both HIV-1 capsid structure and stability. Engineered disulfide crosslinking studies confirmed that the interface is present in the mature viral capsid. The multiple effects of mutations at the interface suggest that small molecules targeting this region of the capsid could be effective antivirals.

## Methods

### Cells and viruses

The 293T, HeLa-P4, HeLa, TZM-bl, and OMK cells used in this study were cultured in Dulbecco’s modification of Eagle’s medium (DMEM; Cellgro) supplemented with 10% fetal bovine serum (FBS), penicillin (50 IU/ml), and streptomycin (50 μg/ml). All cells were cultured in a humidified 37°C incubator containing 5% CO_2_. CEM cells were cultured in RPMI 1640 medium supplemented with 10% FBS, penicillin (50 IU/ml), streptomycin (50 μg/ml). Point mutations in the CA region of the R9 HIV-1 proviral plasmid were introduced by PCR mutagenesis. The mutant viral fragments generated by PCR were reintroduced into R9 with the unique restriction site pairs BssHII-ApaI, Spe1-ApaI, and BssHII-Spe1, depending on the location of the residue on the CA region. The regions of the mutants insert on the viral plasmid corresponding to the PCR-generated fragment were sequenced to confirm the presence of these mutations and absence of extraneous mutations. Viruses used for the abrogation-of-restriction assay were Env-deficient R9 mutants pseudotyped by the vesicular stomatitis virus glycoprotein (VSV-G) [39].

Virus stocks were produced by calcium phosphate transient transfection [54] of 293T cells in which 20 μg of plasmid DNA was used per 2.0 × 10^6^ cells in 10 cm dishes as previously described [10], or proviruses were produced by cotransfection of Env-defective proviral constructs with plasmid pHCMV-G [55]. 12–16 hr after transfection, the culture media was removed, and the cells were washed with sterile PBS before adding fresh media. The culture supernatants were harvested 48 hr after transfection and filtered through a 0.45 μm pore-size filters. Viral aliquots were frozen at −80°C and virus production quantified by p24 ELISA as previously described [56] or by RT activity assay [57].

### Single-cycle assay of HIV-1 infectivity

Viral infectivity was assessed by titration on TZM-bl cells [58]. The TZM-bl cell line is a HeLa cell clone engineered to express CD4+, CXCR-4 and CCR5. These cells contain Tat-responsive luciferase and LacZ reporters. HIV-1 viral stocks were serially diluted in culture medium containing polybrene or DEAE-dextran (8 μg/ml and 20 μg/ml, respectively), and samples (100 μl total volume) were used to inoculate TZM-bl target cells seeded the day before (15,000 cells per well in 96-well plates). The cultures were maintained for an additional 48 hr prior to lysis and detection with a luciferase substrate, (Steady-Glo, Promega). Luminescence in relative light units (RLU) values were quantified in a Topcount instrument (Perkin Elmer). Measurements were normalized by RT or p24 to calculate the relative infectivity of the viruses in RLU/RT or RLU/p24.

### Crosslinking assay

Viruses were pelleted by ultracentrifugation (100,000 ×*g*, 30 min) through a 20% sucrose cushion. The pellets were dissolved in SDS sample buffer in the presence or absence of β-mercaptoethanol. Samples were heated at 95°C for 5 min and then analyzed by SDS-PAGE. CA was detected by immunoblotting using a rabbit polyclonal anti-CA antibody.

### Transmission electron microscopy

Cultured HeLa cells were transfected with 2.5 μg of viral DNA plasmids using the TransIT-HeLaMonster transfection kit (Mirus) according to the manufacturer’s protocol. 24 hr after transfection, cells were washed with phosphate buffered saline (PBS) and fixed with 2.5% glutaraldehyde. The fixed cells were detached from the plates by gentle scraping, pelleted by centrifugation at 4°C and the pellet incubated for 3 hr in an ice-water bath. Following incubation, the cells were washed several times with PBS and the cell pellets were shipped to Electron Microscopy BioServices (Gaithersburg, MD) for embedding, sectioning, and microscopic examination. Pellets were post-fixed in 1% osmium tetroxide and washed in ultra pure water. The samples were then bloc-stained with 2% uranyl acetate and dehydrated in ethanol. Ultra-thin sections were cut and mounted onto 200 mesh copper grids and post-stained with uranyl acetate and Reynolds’s lead citrate; and examined in a FEI Tecnai Spirit Twin Transmission Electron Microscope at 80kV at a minimum magnification of 30,000X. At least 40 different grids from each sample were analyzed, and over 200 particles from random fields of wild type and mutant virions were examined, and a minimum of 10 high-resolution images were acquired for each sample. Between 15 and 40 particles were evaluated for particle diameter by measuring the diameter from prints of the electron micrographs. Measurements were made with a digital vernier caliper and scaled using the size bars on the micrographs.

### Isolation of HIV-1 cores

HIV-1 cores were isolated and characterized as previously described [40]. For each virus, the level of core-associated CA was determined as a percentage of the total CA in the gradient (based on ELISA). We used a commercial ELISA (SAIC, Frederick) to analyze the D166A and R167A mutants, which were not detected by our standard ELISA.

### *In vitro* uncoating assay

The assay was carried out as previously reported [40], with the following modifications. Frozen aliquots of purified HIV-1 cores (300 μl) were thawed and diluted in 300 μl of cold STE buffer. 100 μl of the diluted cores were mixed with 150 μl of 1X STE buffer and incubated at 37°C in a final concentration of 10 μg/ml of BSA. Immediately following incubation, the reaction was stopped by incubation in an ice-water bath for 10 min and then centrifuged at 100,000 ×*g* (Beckman TLA-55 rotor at 45,000 rpm) for 20 min at 4°C to pellet the remaining cores. The supernatant was saved, while the pellet was dissolved in 250 μl of p24 ELISA sample diluent (10% calf serum and 0.5% Triton X-100 in phosphate-buffered saline). The CA content in both fractions (pellet and supernatant) was quantified by p24 ELISA, and the extent of uncoating was determined as the ratio of CA in the supernatant to the total CA quantity in the reaction. It should be noted that variations in wild type uncoating can occur from batch-to-batch preparations of cores, hence wild type and mutant cores shown for each uncoating analysis were isolated at the same time.

### Abrogation-of-restriction assay

The reporter virus, HIV-1-GFP pseudotyped with VSV-G, was used to infect OMK (owl monkey kidney) cells as previously reported [39]. 20,000 cells per well in 12-well plates were seeded 24 hr prior to inoculation. To determine the appropriate dose of the reporter virus to use, the virus was initially titrated on OMK cells. Based on the titration results, we determined the dose of GFP reporter virus to use to achieve an infection level of ~0.5%. The reporter virus together with increasing concentrations of test viruses was inoculated onto OMK cells in 600 μl final volume containing 8 μg/ml polybrene. The inoculum was removed and the medium replenished 24 hr later. 48 hr post-inoculation, cells were dissociated with trypsin and fixed by addition of an equal volume of PBS containing 4% paraformaldehyde. GFP expression was quantified by flow cytometry using an Accuri C6 flow cytometer. The percentage of GFP-expressing cells was determined from a minimum of 10,000 cells analyzed.

### Assay of reverse transcription in target cells

For analysis of viral DNA synthesis by qPCR, viruses were produced in 293T cells using the 293T transfection reagent (Mirus), according to the manufacturer’s protocol. We found this transfection procedure to be effective at for minimizing levels of carryover plasmid DNA, thereby providing acceptable backgrounds in the PCR-based assay of reverse transcription. Briefly, 8 hr post transfection the media was removed and the cells washed with PBS before replenishing media. Viruses were harvested 36–48 hr later. Virus stocks were pretreated with 20 μg of DNase 1, 10 μM MgCl_2_, and 1 μM of CaCl_2_ at 37°C for 1 hr to remove contaminating plasmid DNA, and 100 ng of p24 was used to inoculate 100,000 HeLa-P4 cells per well. To test for potential contaminating plasmid DNA in the virus stocks, infections were performed in the presence of 1 μM Efavirenz, a reverse transcription inhibitor. After 8 hr, cells were harvested and DNA isolated. To harvest the cells, the supernatant was removed and the cells were washed once with 1 ml of PBS and detached with trypsin. Cells were pelleted and washed again with 0.5 ml of PBS and the pellet resuspended in 100 μl PCR lysis buffer (10 mM Tris–HCl, 1 mM EDTA, 0.2 mM CaCl_2_, 0.001% Triton X-100, 0.001% SDS, 1 mg/ml proteinase K) and incubated at 58°C for 1 hr, followed by heat inactivation at 95°C for 15 min. Viral DNA was quantified by real-time PCR using an Stratagene MX-3000p instrument with SYBR Green detection. The reaction mixture contained 5 μl of infected lysate, 12.5 μl of 2X SYBR Green PCR Master Mix (Roche), and 500 nM of each primer. Second strand reverse transcription products were amplified using 5^′^-AGCAGCTGCTTTTTGCCTGTACT-3 as the sense primer and 5^′^-CCTGCGTCGAGAGAGCTCCTCTGG-3^′^, as the reverse primer. The thermal cycling conditions were set at 50°C for 2 min and 95°C for 10 min, followed by 40 cycles of 95°C for 15 s and 65°C for 45 s. We tested how much DNA was loaded to qPCR reactions by measuring the absorbance at 260 nm of the samples, and the quantities of DNA analyzed were within 5% of one another.

## Abbreviations

HIV-1: Human immunodeficiency virus type 1; NTD: Amino-terminal domain; RNP: Ribonucleoprotein; PIC: Preintegration complex; CTD: Carboxy-terminal domain; VLPs: Virus-like particles; VSV-G: Vesicular stomatitis virus glycoprotein; TEM: Transmission electron microscopy; H/D: Hydrogen-deuterium.

## Competing interests

The authors declare that they have no competing interests.

## Authors’ contributions

ELY and CA designed the experiments; ELY performed the experiments and produced the structural figures; ELY and CA interpreted the results and wrote the manuscript. All authors read and approved the final manuscript.

## Authors’ information

This work was performed by ELY in the course of his doctoral research in the laboratory of CA.

## References

[B1] Ganser BK Li S Klishko VY Finch JT Sundquist WI Assembly and analysis of conical models for the HIV-1 core Science 1999 283 80 83 10.1126/science.283.5398.80 9872746

[B2] Ganser-Pornillos BK Yeager M Sundquist WI The structural biology of HIV assembly Curr Opin Struct Biol 2008 18 203 217 10.1016/j.sbi.2008.02.001 18406133 PMC2819415

[B3] Gamble TR Yoo S Vajdos FF von Schwedler UK Worthylake DK Wang H McCutcheon JP Sundquist WI Hill CP Structure of the carboxyl-terminal dimerization domain of the HIV-1 capsid protein Science 1997 278 849 853 10.1126/science.278.5339.849 9346481

[B4] Gitti RK Lee BM Walker J Summers MF Yoo S Sundquist WI Structure of the amino-terminal core domain of the HIV-1 capsid protein Science 1996 273 231 235 10.1126/science.273.5272.231 8662505

[B5] Byeon IJ Meng X Jung J Zhao G Yang R Ahn J Shi J Concel J Aiken C Zhang P Gronenborn AM Structural convergence between Cryo-EM and NMR reveals intersubunit interactions critical for HIV-1 capsid function Cell 2009 139 780 790 10.1016/j.cell.2009.10.010 19914170 PMC2782912

[B6] Ganser-Pornillos BK Cheng A Yeager M Structure of full-length HIV-1 CA: a model for the mature capsid lattice Cell 2007 131 70 79 10.1016/j.cell.2007.08.018 17923088

[B7] Pornillos O Ganser-Pornillos BK Yeager M Atomic-level modelling of the HIV capsid Nature 2011 469 424 427 10.1038/nature09640 21248851 PMC3075868

[B8] Pornillos O Ganser-Pornillos BK Kelly BN Hua Y Whitby FG Stout CD Sundquist WI Hill CP Yeager M X-ray structures of the hexameric building block of the HIV capsid Cell 2009 137 1282 1292 10.1016/j.cell.2009.04.063 19523676 PMC2840706

[B9] Worthylake DK Wang H Yoo S Sundquist WI Hill CP Structures of the HIV-1 capsid protein dimerization domain at 2.6 A resolution Acta Crystallogr D: Biol Crystallogr 1999 55 85 92 10.1107/S0907444998007689 10089398

[B10] Forshey BM von Schwedler U Sundquist WI Aiken C Formation of a human immunodeficiency virus type 1 core of optimal stability is crucial for viral replication J Virol 2002 76 5667 5677 10.1128/JVI.76.11.5667-5677.2002 11991995 PMC137032

[B11] Yang R Aiken C A mutation in alpha helix 3 of CA renders human immunodeficiency virus type 1 cyclosporin A resistant and dependent: rescue by a second-site substitution in a distal region of CA J Virol 2007 81 3749 3756 10.1128/JVI.02634-06 17267487 PMC1866112

[B12] Wacharapornin P Lauhakirti D Auewarakul P The effect of capsid mutations on HIV-1 uncoating Virology 2007 358 48 54 10.1016/j.virol.2006.08.031 16996553

[B13] Hulme AE Perez O Hope TJ Complementary assays reveal a relationship between HIV-1 uncoating and reverse transcription Proc Natl Acad Sci U S A 2011 108 9975 9980 10.1073/pnas.1014522108 21628558 PMC3116424

[B14] Yang R Shi J Byeon IJ Ahn J Sheehan JH Meiler J Gronenborn AM Aiken C Second-site suppressors of HIV-1 capsid mutations: restoration of intracellular activities without correction of intrinsic capsid stability defects Retrovirology 2012 9 30 10.1186/1742-4690-9-30 22515365 PMC3351742

[B15] Sayah DM Sokolskaja E Berthoux L Luban J Cyclophilin A retrotransposition into TRIM5 explains owl monkey resistance to HIV-1 Nature 2004 430 569 573 10.1038/nature02777 15243629

[B16] Stremlau M Owens CM Perron MJ Kiessling M Autissier P Sodroski J The cytoplasmic body component TRIM5alpha restricts HIV-1 infection in Old World monkeys Nature 2004 427 848 853 10.1038/nature02343 14985764

[B17] Stremlau M Perron M Lee M Li Y Song B Javanbakht H Diaz-Griffero F Anderson DJ Sundquist WI Sodroski J Specific recognition and accelerated uncoating of retroviral capsids by the TRIM5alpha restriction factor Proc Natl Acad Sci U S A 2006 103 5514 5519 10.1073/pnas.0509996103 16540544 PMC1459386

[B18] Jiang J Ablan SD Derebail S Hercik K Soheilian F Thomas JA Tang S Hewlett I Nagashima K Gorelick RJ The interdomain linker region of HIV-1 capsid protein is a critical determinant of proper core assembly and stability Virology 2011 421 253 265 10.1016/j.virol.2011.09.012 22036671 PMC3573886

[B19] Noviello CM Lopez CS Kukull B McNett H Still A Eccles J Sloan R Barklis E Second-site compensatory mutations of HIV-1 capsid mutations J Virol 2011 85 4730 4738 10.1128/JVI.00099-11 21367891 PMC3126181

[B20] von Schwedler UK Stray KM Garrus JE Sundquist WI Functional surfaces of the human immunodeficiency virus type 1 capsid protein J Virol 2003 77 5439 5450 10.1128/JVI.77.9.5439-5450.2003 12692245 PMC153941

[B21] Dorfman T Bukovsky A Ohagen A Hoglund S Gottlinger HG Functional domains of the capsid protein of human immunodeficiency virus type 1 J Virol 1994 68 8180 8187 7966609 10.1128/jvi.68.12.8180-8187.1994 PMC237283

[B22] von Schwedler UK Stemmler TL Klishko VY Li S Albertine KH Davis DR Sundquist WI Proteolytic refolding of the HIV-1 capsid protein amino-terminus facilitates viral core assembly EMBO J 1998 17 1555 1568 10.1093/emboj/17.6.1555 9501077 PMC1170503

[B23] Grigorov B Decimo D Smagulova F Pechoux C Mougel M Muriaux D Darlix JL Intracellular HIV-1 Gag localization is impaired by mutations in the nucleocapsid zinc fingers Retrovirology 2007 4 54 10.1186/1742-4690-4-54 17683545 PMC1976323

[B24] Lanman J Sexton J Sakalian M Prevelige PE Jr Kinetic analysis of the role of intersubunit interactions in human immunodeficiency virus type 1 capsid protein assembly *in vitro* J Virol 2002 76 6900 6908 10.1128/JVI.76.14.6900-6908.2002 12072491 PMC136311

[B25] Lanman J Lam TT Barnes S Sakalian M Emmett MR Marshall AG Prevelige PE Jr Identification of novel interactions in HIV-1 capsid protein assembly by high-resolution mass spectrometry J Mol Biol 2003 325 759 772 10.1016/S0022-2836(02)01245-7 12507478

[B26] Bowzard JB Wills JW Craven RC Second-site suppressors of Rous sarcoma virus Ca mutations: evidence for interdomain interactions J Virol 2001 75 6850 6856 10.1128/JVI.75.15.6850-6856.2001 11435564 PMC114412

[B27] Cardone G Purdy JG Cheng N Craven RC Steven AC Visualization of a missing link in retrovirus capsid assembly Nature 2009 457 694 698 10.1038/nature07724 19194444 PMC2721793

[B28] Inagaki N Takeuchi H Yokoyama M Sato H Ryo A Yamamoto H Kawada M Matano T A structural constraint for functional interaction between N-terminal and C-terminal domains in simian immunodeficiency virus capsid proteins Retrovirology 2010 7 90 10.1186/1742-4690-7-90 20955553 PMC2964592

[B29] Berthet-Colominas C Monaco S Novelli A Sibai G Mallet F Cusack S Head-to-tail dimers and interdomain flexibility revealed by the crystal structure of HIV-1 capsid protein (p24) complexed with a monoclonal antibody Fab EMBO J 1999 18 1124 1136 10.1093/emboj/18.5.1124 10064580 PMC1171204

[B30] Khorasanizadeh S Campos-Olivas R Summers MF Solution structure of the capsid protein from the human T-cell leukemia virus type-I J Mol Biol 1999 291 491 505 10.1006/jmbi.1999.2986 10438634

[B31] Momany C Kovari LC Prongay AJ Keller W Gitti RK Lee BM Gorbalenya AE Tong L McClure J Ehrlich LS Crystal structure of dimeric HIV-1 capsid protein Nat Struct Biol 1996 3 763 770 10.1038/nsb0996-763 8784350

[B32] Kingston RL Fitzon-Ostendorp T Eisenmesser EZ Schatz GW Vogt VM Post CB Rossmann MG Structure and self-association of the Rous sarcoma virus capsid protein Structure 2000 8 617 628 10.1016/S0969-2126(00)00148-9 10873863

[B33] Ako-Adjei D Johnson MC Vogt VM The retroviral capsid domain dictates virion size, morphology, and coassembly of gag into virus-like particles J Virol 2005 79 13463 13472 10.1128/JVI.79.21.13463-13472.2005 16227267 PMC1262573

[B34] Lanman J Lam TT Emmett MR Marshall AG Sakalian M Prevelige PE Jr Key interactions in HIV-1 maturation identified by hydrogen-deuterium exchange Nat Struct Mol Biol 2004 11 676 677 10.1038/nsmb790 15208693

[B35] Bartonova V Igonet S Sticht J Glass B Habermann A Vaney MC Sehr P Lewis J Rey FA Krausslich HG Residues in the HIV-1 capsid assembly inhibitor binding site are essential for maintaining the assembly-competent quaternary structure of the capsid protein J Biol Chem 2008 283 32024 32033 10.1074/jbc.M804230200 18772135

[B36] Joshi A Nagashima K Freed EO Mutation of dileucine-like motifs in the human immunodeficiency virus type 1 capsid disrupts virus assembly, gag-gag interactions, gag-membrane binding, and virion maturation J Virol 2006 80 7939 7951 10.1128/JVI.00355-06 16873251 PMC1563813

[B37] Scholz I Arvidson B Huseby D Barklis E Virus particle core defects caused by mutations in the human immunodeficiency virus capsid N-terminal domain J Virol 2005 79 1470 1479 10.1128/JVI.79.3.1470-1479.2005 15650173 PMC544128

[B38] Brun S Solignat M Gay B Bernard E Chaloin L Fenard D Devaux C Chazal N Briant L VSV-G pseudotyping rescues HIV-1 CA mutations that impair core assembly or stability Retrovirology 2008 5 57 10.1186/1742-4690-5-57 18605989 PMC2474847

[B39] Forshey BM Shi J Aiken C Structural requirements for recognition of the human immunodeficiency virus type 1 core during host restriction in owl monkey cells J Virol 2005 79 869 875 10.1128/JVI.79.2.869-875.2005 15613315 PMC538572

[B40] Shah VB Aiken C *In vitro* uncoating of HIV-1 cores J Vis Exp 2011 57 e3384 10.3791/3384 PMC3308611 22105356

[B41] Shi J Zhou J Shah VB Aiken C Whitby K Small-molecule inhibition of human immunodeficiency virus type 1 infection by virus capsid destabilization J Virol 2011 85 542 549 10.1128/JVI.01406-10 20962083 PMC3014163

[B42] Diaz-Griffero F Kar A Lee M Stremlau M Poeschla E Sodroski J Comparative requirements for the restriction of retrovirus infection by TRIM5alpha and TRIMCyp Virology 2007 369 400 410 10.1016/j.virol.2007.08.032 17920096 PMC2153441

[B43] Diaz-Griffero F Kar A Perron M Xiang SH Javanbakht H Li X Sodroski J Modulation of retroviral restriction and proteasome inhibitor-resistant turnover by changes in the TRIM5alpha B-box 2 domain J Virol 2007 81 10362 10378 10.1128/JVI.00703-07 17626085 PMC2045480

[B44] Diaz-Griffero F Vandegraaff N Li Y McGee-Estrada K Stremlau M Welikala S Si Z Engelman A Sodroski J Requirements for capsid-binding and an effector function in TRIMCyp-mediated restriction of HIV-1 Virology 2006 351 404 419 10.1016/j.virol.2006.03.023 16650449

[B45] Shi J Aiken C Saturation of TRIM5 alpha-mediated restriction of HIV-1 infection depends on the stability of the incoming viral capsid Virology 2006 350 493 500 10.1016/j.virol.2006.03.013 16624363

[B46] Leschonsky B Ludwig C Bieler K Wagner R Capsid stability and replication of human immunodeficiency virus type 1 are influenced critically by charge and size of Gag residue 183 J Gen Virol 2007 88 207 216 10.1099/vir.0.81894-0 17170453

[B47] Forshey BM Aiken C Disassembly of human immunodeficiency virus type 1 cores *in vitro* reveals association of Nef with the subviral ribonucleoprotein complex J Virol 2003 77 4409 4414 10.1128/JVI.77.7.4409-4414.2003 12634398 PMC150647

[B48] Meng X Zhao G Yufenyuy E Ke D Ning J Delucia M Ahn J Gronenborn AM Aiken C Zhang P Protease Cleavage Leads to Formation of Mature Trimer Interface in HIV-1 Capsid PLoS Pathog 2012 8 e1002886 10.1371/journal.ppat.1002886 22927821 PMC3426514

[B49] Kelly BN Kyere S Kinde I Tang C Howard BR Robinson H Sundquist WI Summers MF Hill CP Structure of the antiviral assembly inhibitor CAP-1 complex with the HIV-1 CA protein J Mol Biol 2007 373 355 366 10.1016/j.jmb.2007.07.070 17826792 PMC2066180

[B50] Kortagere S Madani N Mankowski MK Schon A Zentner I Swaminathan G Princiotto A Anthony K Oza A Sierra LJ Inhibiting Early-Stage Events in HIV-1 Replication by Small-Molecule Targeting of the HIV-1 Capsid J Virol 2012 86 8472 8481 10.1128/JVI.05006-11 22647699 PMC3421734

[B51] Lemke CT Titolo S von Schwedler U Goudreau N Mercier JF Wardrop E Faucher AM Coulombe R Banik SS Fader L Distinct effects of two HIV-1 capsid assembly inhibitor families that bind the same site within the N-terminal domain of the viral CA protein J Virol 2012 86 6643 6655 10.1128/JVI.00493-12 22496222 PMC3393593

[B52] Blair WS Pickford C Irving SL Brown DG Anderson M Bazin R Cao J Ciaramella G Isaacson J Jackson L HIV capsid is a tractable target for small molecule therapeutic intervention PLoS Pathog 2010 6 e1001220 10.1371/journal.ppat.1001220 21170360 PMC3000358

[B53] Tang C Loeliger E Kinde I Kyere S Mayo K Barklis E Sun Y Huang M Summers MF Antiviral inhibition of the HIV-1 capsid protein J Mol Biol 2003 327 1013 1020 10.1016/S0022-2836(03)00289-4 12662926

[B54] Chen C Okayama H High-efficiency transformation of mammalian cells by plasmid DNA Mol Cell Biol 1987 7 2745 2752 3670292 10.1128/mcb.7.8.2745-2752.1987 PMC367891

[B55] Yee JK Friedmann T Burns JC Generation of high-titer pseudotyped retroviral vectors with very broad host range Methods Cell Biol 1994 43 99 112 7823872 10.1016/s0091-679x(08)60600-7

[B56] Wehrly K Chesebro B p24 antigen capture assay for quantification of human immunodeficiency virus using readily available inexpensive reagents Methods 1997 12 288 293 10.1006/meth.1997.0481 9245608

[B57] Aiken C Pseudotyping human immunodeficiency virus type 1 (HIV-1) by the glycoprotein of vesicular stomatitis virus targets HIV-1 entry to an endocytic pathway and suppresses both the requirement for Nef and the sensitivity to cyclosporin A J Virol 1997 71 5871 5877 9223476 10.1128/jvi.71.8.5871-5877.1997 PMC191842

[B58] Wei X Decker JM Liu H Zhang Z Arani RB Kilby JM Saag MS Wu X Shaw GM Kappes JC Emergence of resistant human immunodeficiency virus type 1 in patients receiving fusion inhibitor (T-20) monotherapy Antimicrob Agents Chemother 2002 46 1896 1905 10.1128/AAC.46.6.1896-1905.2002 12019106 PMC127242

